# Berberine-sonodynamic therapy induces autophagy and lipid unloading in macrophage

**DOI:** 10.1038/cddis.2016.354

**Published:** 2017-01-19

**Authors:** Jiayuan Y Kou, Ying Li, Zhaoyu Y Zhong, Yueqing Q Jiang, Xuesong S Li, Xiaobo B Han, Zhongni N Liu, Ye Tian, Liming M Yang

**Affiliations:** 1 Department of Pathophysiology, Key Laboratory of Cardiovascular Pathophysiology, Harbin Medical University, Harbin, PR China; 2 Department of Oncology, The First Affiliated Clinic College of Harbin Medical University, Harbin, PR China; 3 Division of Cardiology, The First Affiliated Hospital, Harbin Medical University, Harbin, PR China

## Abstract

Impaired autophagy in macrophages accompanies the progression of atherosclerosis and contributes to lipid loading in plaques and ineffective lipid degradation. Therefore, evoking autophagy and its associated cholesterol efflux may provide a therapeutic treatment for atherosclerosis. In the present study, berberine-mediated sonodynamic therapy (BBR-SDT) was used to induce autophagy and cholesterol efflux in THP-1 macrophages and derived foam cells. Following BBR-SDT, autophagy was increased in the macrophages, autophagy resistance in the foam cells was prevented, and cholesterol efflux was induced. The first two effects were blocked by the reactive oxygen species scavenger, N-acetyl cysteine. BBR-SDT also reduced the phosphorylation of Akt and mTOR, two key molecules in the PI3K/AKT/mTOR signaling pathway, which is responsible for inducing autophagy. Correspondingly, treatment with the autophagy inhibitor, 3-methyladenine, or the PI3K inhibitor, LY294002, abolished the autophagy-induced effects of BBR-SDT. Furthermore, induction of cholesterol efflux by BBR-SDT was reversed by an inhibition of autophagy by 3-methyladenine or by a small interfering RNA targeting *Atg5*. Taken together, these results demonstrate that BBR-SDT effectively promotes cholesterol efflux by increasing reactive oxygen species generation, and this subsequently induces autophagy via the PI3K/AKT/mTOR signaling pathway in both ‘normal' macrophages and lipid-loaded macrophages (foam cells). Thus, BBR-SDT may be a promising atheroprotective therapy to inhibit the progression of atherosclerosis and should be further studied.

Atherosclerosis is a lipoprotein-driven disease that leads to plaque formation in the vasculature, and its progression increases the risk of acute coronary events.^1, 2, 3^ Monocytes/macrophages play critical roles throughout the process of atherogenesis by up taking modified forms of low-density lipoprotein (LDL). This uptake facilitates the transition of LDL into arterial foam cells, which is an essential step in the development of atherosclerotic plaques.^4, 5, 6, 7^ Degradation of lipoprotein in foam cells would prevent the progression of atherosclerosis.^8, 9^ However, deficiencies in cholesterol metabolism and cholesterol efflux have been associated with atherogenesis plaques, thereby leading to aberrant lipid management and enhanced plaque formation. Therefore, the ability to enhance cholesterol efflux from macrophages and foam cells is considered atheroprotective.^10, 11^

Autophagy is a double membrane-driven process that involves trafficking of the cytoplasm to lysosomes for degradation and recycling. As a result, autophagy contributes to the metabolism of proteins, glucose, and lipids.^12, 13, 14^ A growing body of evidence suggests that autophagic dysregulation is associated with atherogenesis progression.^15, 16^ Under atherogenic conditions, induction of autophagy in macrophages contributes to the intracellular breakdown of lipids and the delivery of hydrolyzed cholesterol esters to lysosomes for cholesterol efflux.^17^ However, in atherosclerotic plaques, autophagy is compromised and it contributes to the storage of excess lipids and insufficient lipid degradation.^18, 19, 20, 21^

Considerable effort has been devoted to identifying novel and reliable atherosclerosis therapies. Sonodynamic therapy (SDT) has recently been applied to cancer, as well as atherosclerosis,^22-25^ [Bibr bib25]and therapeutic benefits have been observed in both sets of patients (with the latter observations in progress in our group).^26^ Other studies^27-29^ have identifiedreactive oxygen species (ROS) as a key factor in mediating the biological effects induced by SDT. ROS have also been shown to induce autophagy.^30, 31^ Moreover, SDT-induced autophagy has been observed in many cell types,^32-37^ although itspotential role in macrophages and/or foam cells has not been extensively studied.

The selection of a sonosensitizer has been found to be important for achieving sonodynamic activity. In recent years, our group has identified sonosensitizers, in particular, Chinese herbs have been found to play an important role in SDT in macrophages.^29, 38-40^ For the clinical application of SDT, a water-soluble sonosensitizer is needed. Berberine is a molecule that has been isolated from *Coptischinensis*, and commonly used to treat diarrhea in China. Berberine has also been found to effect obesity, hyperglycemia, and dyslipidemia.^41^ In the present study, the effects of using berberine to trigger ultrasound activation, referred to as berberine-mediated SDT (BBR-SDT), was examined. In particular, the role of BBR-SDT in mediating an induction of autophagy and cholesterol efflux via ROS generation in THP-1 macrophages, peritoneal macrophages, and derived foam cells was studied.

## Results

### Berberine acts as a sonosensitizer to induce autophagy in macrophages upon exposure to ultrasound

**Figure 1:**
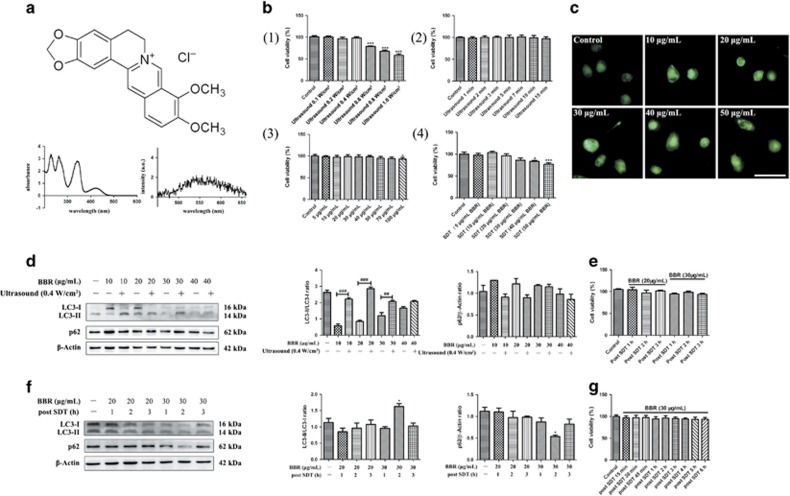
The sonosensitizer, berberine, induced autophagy of macrophages upon ultrasound exposure. (**a**) The chemical structure, absorption spectrum, and fluorescence emission spectrum of berberine (dissolved in ddH_2_O) are shown. (**b**) The effects of berberine with or without ultrasound irradiation on the viability of THP-1 macrophages with the application of: (1) different ultrasound intensities, (2) varying durations of ultrasound exposure (0.4 W/cm^2^), (3) different concentrations of berberine, and (4) different concentrations of BBR-SDT (0.4 W/cm^2^ ultrasound irradiation) as indicated. Cell viability was analyzed in CCK-8 assays and the data are presented as the mean±S.D. (*P<0.05, ***P<0.001 *versus* control). (**c**) Autophagic vacuoles in BBR-SDT-treated cells were coated with varying concentrations of MDC (as indicated) for 30 min. Representative images of THP-1 macrophages are shown 2 h post-BBR-SDT. Scale bar, 50 *μ*m. (**d**) THP-1 macrophages were treated with the indicated concentrations of berberine for 4 h in duplicate, and then one sample of each concentration was exposed to ultrasound. LC3-I, LC3-II, and p62 were detected by western blot and quantifications of the data are shown in the right two panels (*n*=3; ^##^*P*<0.01, ^###^*P*<0.001 *versus* BBR groups). Two specific concentrations of BBR-SDT (20 or 30 *μ*g/ml) were then applied for 1, 2, or 3 h and cell viability and protein levels of LC3-I, LC3-II, and p62 were analyzed in CCK-8 assays (**e**) and western blots (**f**), respectively. The data are quantified in the two panels to the right with mean±S.D. values presented (*n*=3; *P<0.05 versus control). (**g**) Cell viability of THP-1 macrophages was assessed at various time points post-BBR-SDT (30 *μ*g/ml), as indicated, in CCK-8 assays

Our previous studies and those by other groups have applied different sonosensitizers to various cell lines for SDT. The effects have ranged from cell survival to cell death. Figure 1a shows the chemical structure, absorption spectrum, and fluorescence emission spectrum for the sonosensitizer, berberine hydrochloride (dissolved in water). To ensure that the application of ultrasound alone did not affect cell survival, the intensity and exposure time of the ultrasound applied was examined. In these experiments, cell viability was decreased from 0.6 W/cm^2^ and higher (Figure 1b (1)), and remained stable at 0.4 W/cm^2^ with increasing durations of exposure (Figure 1b (2)). A 10-min exposure time at 0.4 W/cm^2^ intensity was selected for the SDT that was applied to the macrophages in this study. When cell viability was analyzed following the treatment of macrophages with various doses of berberine (Figure 1b (3) and Supplementary Figure S1a (1)), no significant decrease was observed until the concentrations of berberine exceeded 100 *μ*g/ml. However, cell viability did decrease at concentrations of berberine greater than 40 *μ*g/ml when ultrasound irradiation was also applied (Figure 1b (4) and Supplementary Figure S1a (2)). Thus, the dose of berberine that was applied to the macrophages in this study was less than 40 *μ*g/ml.

To test whether BBR-SDT induces autophagy in macrophages, autophagic flux induced by BBR-SDT was detected with an autofluorescent substance, monodansylcadaverine (MDC), which is used as a marker of autophagic vacuoles (AVs).^42^ Under a fluorescence microscope, AVs stained by MDC appeared as distinct dot-like structures. MDC staining further showed that the number of strongly stained cells with punctate spots was significantly enhanced in the BBR-SDT-treated cells compared with the control cells, with the latter exhibiting diffuse MDC staining throughout the cytoplasm with very few punctate spots (Figure 1c). However, differences in punctate staining between the BBR-SDT groups were less distinct. Western blotting showed that the ratio of LC3-II/LC3-I increased in a dose-dependent manner in the BBR-SDT-treated macrophages (Figure 1d and Supplementary Figure S1b). The LC3-II/LC3-I ratio was greater in the BBR-SDT groups than in BBR alone groups, suggesting that BBR and ultrasound irradiation have a synergistic effect on autophagy compared with BBR alone. Meanwhile, no obvious cell damage was observed 1, 2, or 3 h post-BBR-SDT (Figure 1e). Western blotting analysis also demonstrated that an increase in the LC3-II/LC3-I ratio was accompanied by a decrease in p62 following 30 *μ*g/ml BBR-SDT (Figure 1f). Meanwhile, no obvious changes in cell survival were observed (Figure 1g). Hence, 30 *μ*g/ml was the concentration of berberine that was selected for use in BBR-SDT of the macrophages.

### Autophagy in macrophages is triggered by BBR-SDT in a time-dependent manner

**Figure 2:**
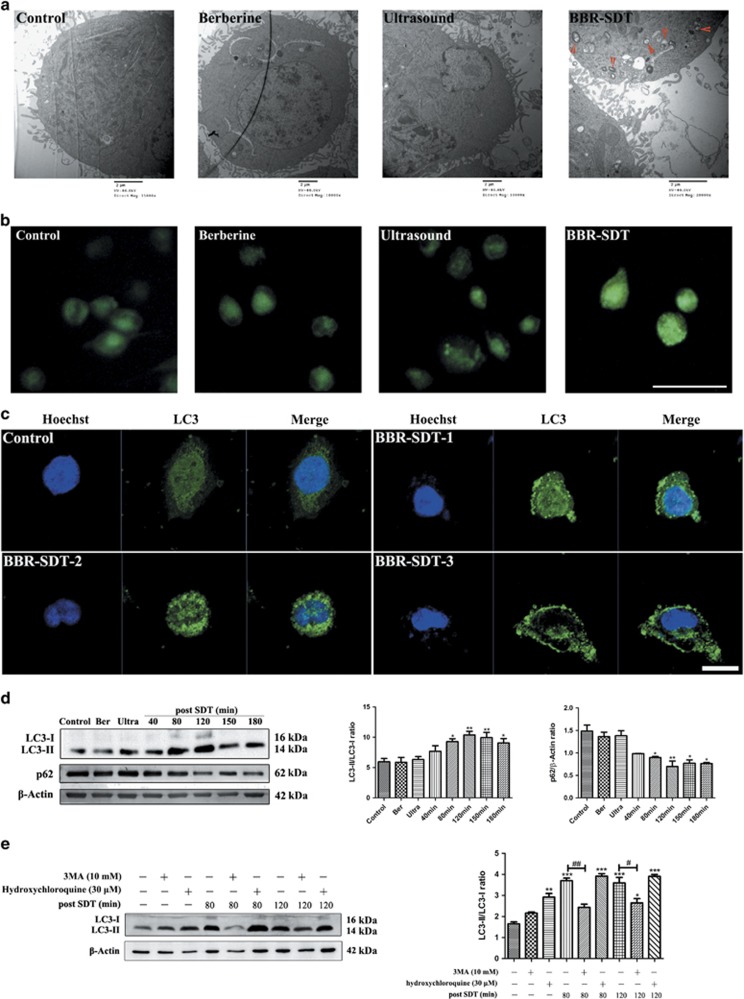
Autophagy triggered by BBR-SDT in a time-dependent manner in THP-1 macrophages. (**a**) Ultrastructural changes in untreated (control), berberine-treated, ultrasound-treated, and BBR-SDT-treated THP-1 macrophages were observed by TEM at 120 min post-BBR-SDT. Red arrows indicate autophagosomes. (**b**) THP-1 macrophages in the control, berberine, ultrasound, and BBR-SDT groups were incubated with MDC (50 *μ*M) for 30 min. Scale bar, 50 *μ*m. (**c**) THP-1 macrophages were stained with an anti-LC3 antibody and Hoechst 33358 at 1, 2, and 3 h post-BBR-SDT. White arrows indicate punctate spots of LC3. Scale bar, 25 *μ*m. (**d**) Expression levels of LC3-I, LC3-II, and p62 were analyzed by western blotting for untreated (control), berberine-treated (Ber), ultrasound-treated (Ultra), and SDT-treated THP-1 macrophages at the indicated time points. The data are quantified in the two panels to the right with mean±S.D. values presented (*n*=3; *P<0.05, **P<0.01 *versus* control). (**e**) Expression levels of LC3-I and LC3-II were analyzed by western blotting at 80 and 120 min following BBR-SDT with or without 3MA- and hydroxychloroquine-pretreatments. Quantification of the LC3-II/LC3-I ratios are shown (*n*=3; *P<0.05, **P<0.01, ***P<0.001 versus control, ^#^*P*<0.05, ^##^*P*<0.01 *versus* BBR-SDT groups)

To further identify the autophagy-inducing effects of BBR-SDT in macrophages, transmission electron microscopy (TEM) and MDC staining were performed. TEM following BBR-SDT showed autophagosomes with a distinct double membrane structure present, and this structure was either absent or rare in the control cells (Figure 2a and Supplementary Figure S1c). For the MDC stainings, an increase in MDC-labeled vesicles was observed for the BBR-SDT group compared with the control groups (Figure 2b).

When BBR-SDT-treated macrophages were stained with an anti-LC3 antibody, a greater numbers of LC3-stained punctate spots were observed in the BBR-SDT groups than in the control groups. To further validate these results, the conversion of LC3-I to LC3-II and degradation of p62 post-BBR-SDT were examined (Figure 2d and Supplementary Figure S1d). The ratio of LC3-II/LC3-I increased in a time-dependent manner post-BBR-SDT, while the levels of p62 decreased. When hydroxychloroquine, a known inhibitor of autophagosome–lysosome fusion, was included in these assays, LC3-II levels increased as a result of the blockage of LC3-II degradation. Moreover, 3-methyladenine (3MA), a widely used autophagy inhibitor, suppressed the BBR-SDT-induced LC3 conversion post-BBR-SDT (Figure 2e and Supplementary Figure S1e).

### Autophagy triggered by BBR-SDT is suppressed by the ROS scavenger, N-acetyl cysteine (NAC)

**Figure 3:**
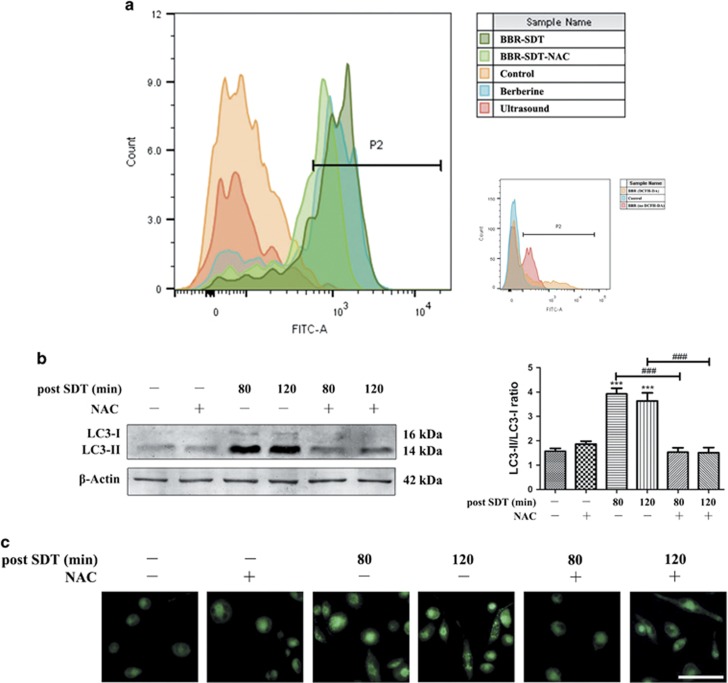
Autophagy triggered by BBR-SDT is suppressed by the ROS scavenger, NAC. (**a**) The relative fluorescence intensity for ROS generation detected in THP-1 macrophages with or without pretreatment with the ROS scavenger, NAC (1 mM), as determined by flow cytometry following DCFH-DA staining (*n*=3; berberine autofluorescence). (**b**) Detection of LC3-I and LC3-II protein levels in THP-1 macrophages with or without NAC-pretreatment at 80 and 120 min post-BBR-SDT by western blotting. Quantitation of the LC3-II/LC3-I ratios are presented to the right as the mean±S.D. (*n*=3; ***P<0.001 *versus* control, ^###^*P*<0.001 *versus* BBR-SDT groups). (**c**) Autophagic vacuoles were detected in THP-1 macrophages that were incubated with MDC (50 *μ*M) for 30 min. Scale bar, 50 *μ*m.

Induction of ROS is considered to be the primary mechanism mediated by SDT.^43, 44^ Therefore, production of ROS was analyzed with dichloro-dihydro-fluorescein diacetate (DCFH-DA) staining by flow cytometry. The levels of ROS increased following BBR-SDT compared with the other control groups (Figure 3a and Supplementary Figure S1f). Conversely, treatment of the macrophages with the ROS scavenger, NAC, effectively blocked the increase in ROS that was induced by BBR-SDT.

To investigate whether the autophagy induced by BBR-SDT was associated with the generation of ROS, the LC3-II/LC3-I ratio post-BBR-SDT was examined for macrophages that were pretreated with NAC. In the western blot analysis performed, no significant difference in LC3-II/LC3-I ratio between the BBR-SDT group pretreated with NAC and the control groups were observed (Figure 3b and Supplementary Figure S1e). These results indicate that NAC is able to partially prevent an increase in LC3 conversion that is induced by BBR-SDT. Moreover, there was no obvious enhancement in autophagy following the scavenging of ROS. Similar effects of BBR-SDT on ROS generation and autophagy induction were also indicated by MDC staining (Figure 3c).

### BBR-SDT induces autophagy by inhibiting PI3K/AKT/mTOR signaling in macrophages

**Figure 4:**
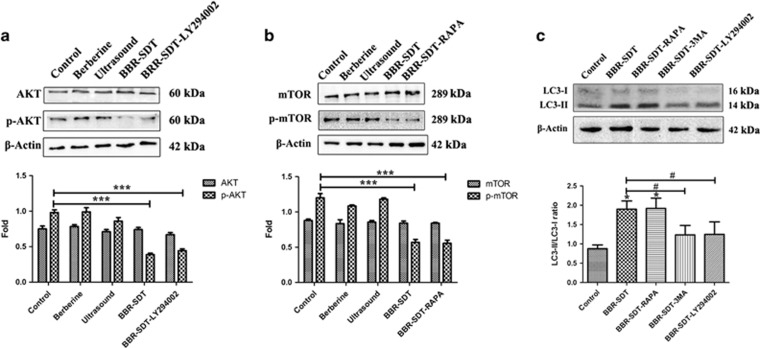
BBR-SDT induces autophagy by inhibiting PI3K/AKT/mTOR signaling in macrophages. (**a**) Total protein extracts from untreated THP-1 macrophages (control) and macrophages treated with berberine, ultrasound, BBR-SDT, or 5 *μ*M LY294002 or 1 *μ*M rapamycin (RAPA) before BBR-SDT were analyzed in western blots to detect (**a**) AKT and p-AKT (Ser 473) and (**b**) mTOR and p-mTOR (Ser 2448), respectively. Fold changes in protein expression are provided in the bar graphs below each set of blots. (*n*=3; ***P<0.001 *versus* control). (**c**) Levels of LC3-I and LC3-II in extracts of THP-1 macrophages post-BBR-SDT after pretreatment with rapamycin (RAPA), 3MA (10 mM), or LY294002. Quantifications of the LC3-II/LC3-I ratios are presented below (*n*=3; *P<0.05 *versus* control, ^#^*P*<0.05 *versus* BBR-SDT groups)

Regulation of autophagy involves mTOR as an upstream signal, and this protein is positively regulated by PI3K/Akt to inhibit autophagy. To investigate whether mTOR-mediated signaling is involved in BBR-SDT-induced autophagy, the total and phosphorylated levels of AKT and mTOR were detected following BBR-SDT. As shown in Figures 4a and b, the levels of total AKT and mTOR were not affected by BBR-SDT, whereas the levels of Akt phosphorylation at Ser 473 and mTOR phosphorylation at Ser 2448 were significantly decreased post-BBR-SDT. Similar decreases in the levels of p-AKT and p-mTOR were observed following the administration of PI3K and mTOR inhibitors, LY294002 and rapamycin, respectively. Following LY294002 and rapamycin were applied to macrophages before BBR-SDT treatment, results showed that the BBR-SDT-induced LC3 conversion was reduced by LY294002, and was increased by rapamycin (Figure 4c). These results imply that p-AKT and p-mTOR are key molecules involved in BBR-SDT-induced autophagy in macrophages.

### BBR-SDT-induced autophagy in autophagy-resistant foam cells that were induced by ox-LDL

**Figure 5:**
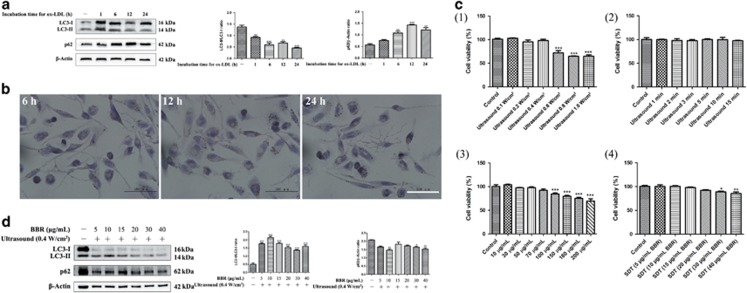
BBR-SDT similarly induced autophagy in autophagy-resistant foam cells induced by ox-LDL. (**a**) THP-1 macrophages were incubated with ox-LDL for various periods of time as indicated. Levels of LC3-I, LC3-II, and p62 were then analyzed by western blotting and the data are quantified in the two panels to the right (*n*=3; **P<0.01 and ***P<0.001 *versus* control). (**b**) Representative images of THP-1 macrophage morphology following incubation with ox-LDL for 6, 12, and 24 h are shown. Oil Red O staining was performed to examine foam cell formation. (**c**) Cell viability was assayed in CCK-8 assays for foam cells that were treated with: (1) different ultrasound intensities, (2) varying durations of exposure to ultrasound (0.4 W/cm^2^), (3) different concentrations of berberine, and (4) different concentrations of BBR-SDT (0.4 W/cm^2^) (*P<0.05, **P<0.01, and ***P<0.001 *versus* control). (**d**) Levels of LC3-I, LC3-II, and p62 in foam cells post-BBR-SDT were analyzed by western blotting. Quantitation of the LC3-II/LC3-I ratios are presented to the right as the mean±S.D. (*n*=3; *P<0.05, **P<0.01, and ***P<0.001 *versus* control)

Next, autophagy was examined in macrophages that were exposed to ox-LDL. These macrophages exhibited a decrease in LC3-II/LC3-I ratio and an increase in the levels of p62 in a time-dependent manner (Figure 5a). Furthermore, these effects were most obvious after 6 h of exposure to ox-LDL, implying that autophagy resistance was observed. To investigate the role of BBR-SDT in an autophagy impaired cell line, a 12-h incubation time with ox-LDL was applied to foam cells. This period of time was selected based on the results described above and other related studies.^43, 44^ Foam cell formation was observed in Oil Red O assays after macrophages were exposed to ox-LDL (Figure 5b and Supplementary Figure S2a).

After establishing an autophagy-resistant model with foam cells, ultrasonic parameters and berberine doses were re-optimized. For this, cell viability was analyzed following the application of different ultrasound parameters and different doses of berberine to the foam cells (Figure 5c and Supplementary Figure S2b (1)). Collectively, these results indicate that ultrasound parameters that were applied to the macrophages were also suitable for the foam cells (Supplementary Figure S2b (2)). In Figure 5d and Supplementary Figure S2c, the data show that the ratio of LC3-II/LC3-I increased, whereas the levels of p62 decreased, in a dose-dependent manner. Moreover, an obvious effect was observed with application of 10 *μ*g/ml BBR-SDT in THP-1 macrophage-derived foam cells and 20 *μ*g/ml BBR-SDT in peritoneal macrophage-derived foam cells (Supplementary Figure S2d).

### BBR-SDT-induced autophagy and suppression of PI3K/AKT/mTOR signaling in foam cells are time dependent

**Figure 6:**
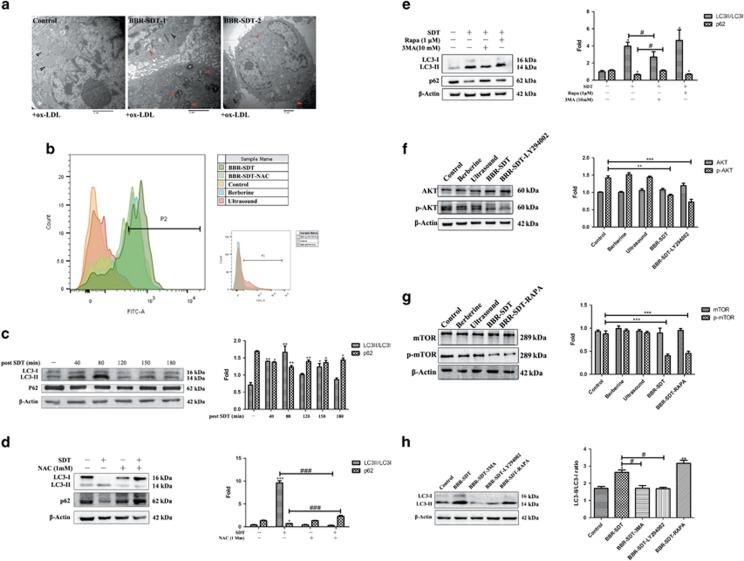
Time-dependent induction of autophagy and suppressed PI3K/AKT/mTOR signaling in foam cells treated with BBR-SDT. (**a**) Ultrastructural changes in foam cells treated with ox-LDL were observed by TEM at 1 and 2 h post-BBR-SDT. Black arrows indicate lipid droplets, red arrows indicate autophagosomes. Scale bar, 2 *μ*m.(**b**) The relative fluorescence intensity for ROS generation detected in foam cells with or without pretreatment with the ROS scavenger, NAC (1 mM), as determined by flow cytometry following DCFH-DA staining (*n*=3; berberine autofluorescence). Western blot analysis and quantification of fold change in LC3-II/LC3-I ratios and p62 levels in foam cells: (**c**) post-BBR-SDT at different time points (*P<0.05 and **P<0.01 *versus* control), (**d**) with and without pretreatment with NAC and post-BBR-SDT (*P<0.05 and ***P<0.001 *versus* control; ^###^*P*<0.001 *versus* BBR-SDT groups), and (**e**) with and without rapamycin (Rapa) and 3MA pretreatment and post-BBR-SDT (*P<0.05 *versus* control, ^#^*P*<0.05 *versus* BBR-SDT groups). In addition, western blots were analyzed post-BBR-SDT for: (**f**) levels of total Akt and p-Akt following LY294002 pretreatment, (**g**) levels of total mTOR and p-mTOR following rapamycin (RAPA) pretreatment, and (**h**) levels of LC3-I, LC3-II, and *β*-actin following pretreatment with LY294002, 3MA, or rapamycin (RAPA). The corresponding quantifications of fold change and ratios are presented as the mean±S.D. (*P<0.05, **P<0.01, and ***P<0.001 *versus* control, ^#^*P*<0.05 *versus* BBR-SDT groups)

Obvious morphologic changes were observed in the BBR-SDT-treated foam cells compared with the control cells (Figure 6a and Supplementary Figure S2e), and these included an increase in the number of autophagosomes and autolysosomes and a decrease in the number of lipid droplets post-BBR-SDT. In addition, an increase in the amount of ROS was detected following BBR-SDT (Figure 6b and Supplementary Figure S2f). The ROS scavenger, NAC, was also found to effectively block BBR-SDT-induced ROS generation. The data in Figure 6c show that autophagy resistance induced by ox-LDL was markedly reversed by BBR-SDT in a time-dependent manner, based on the increase in the LC3-II/LC3-I ratio and the decrease in p62 levels that were observed. Moreover, the maximal effect was observed at 80 min post-BBR-SDT. Treatment with NAC exerted the opposite effect (Figure 6d and Supplementary Figure S2g), thereby indicating that ROS are involved in BBR-SDT-induced autophagy in foam cells. Western blotting further showed that rapamycin rescued the autophagy process in foam cells similar to the autophagy induced by BBR-SDT, whereas 3MA abolished this effect (Figure 6e and Supplementary Figure S2g).

The levels of total AKT and mTOR were not affected by BBR-SDT, whereas the levels of p-AKT and p-mTOR were significantly decreased post-BBR-SDT (Figure 6f). Similar results were observed following the treatments with LY294002 and rapamycin (Figure 6g). In Figure 6 h, the BBR-SDT-induced LC3 conversion was shown to decrease following LY294002 treatment, whereas it increased following rapamycin treatment.

### Blockage of autophagy reversed BBR-SDT-induced autophagy and cholesterol efflux in foam cells

**Figure 7:**
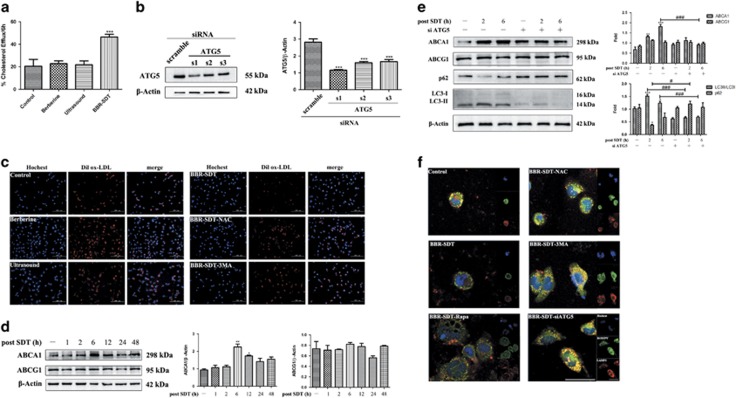
Blockage of autophagy reverses BBR-SDT-induced autophagy and cholesterol efflux in foam cells. (**a**) Macrophages were incubated with labeling media and then were treated with various HDL (50 *μ*g) as cholesterol acceptors to induce cholesterol efflux post-BBR-SDT. The data are expressed as % efflux detected 6 h post-BBR-SDT (*n*=3; ***P<0.001 versus control). (**b**) Representative western blot and quantification of ATG5 levels following siRNA treatment (*n*=3; ^***^P<0.001 *versus* control). (**c**) Fluorescence microscopy images of macrophages that were pretreated with 3MA or NAC, were subjected to BBR-SDT, and then were incubated with Dilox-LDL for 6 h. Red: Dilox-LDL derivatives, blue: Hoechst-stained nuclei. (**d**) At various time points post-BBR-SDT, extracts of foam cells were subjected to western blotting to analyze levels of ABCA1, ABCG1, and *β*-actin. Quantification of these levels are shown to the right as mean±S.D. values (*n*=3; *P<0.05 and **P<0.01 *versus* control). (**e**) Western blots were analyzed for levels of ABCA1, ABCG1, p62, LC3-I, LC3-II, and *β*-actin in foam cell extracts at various time points following BBR-SDT as indicated with and without siATG5 pretreatment. Quantification of the fold change for these proteins are shown to the right (*n*=3; *P<0.05,**P<0.01, and ***P<0.001 *versus* control; ^#^*P*<0.05 and ^###^*P*<0.001 *versus* BBR-SDT groups). (**f**) Lipid droplets and lysosomes were visualized in foam cells 6 h after BBR-SDT with and without rapamycin (Rapa), NAC, 3MA, and siATG5 treatments with stainings performed with Bodipy FL (0.1 *μ*M) and antibodies raised against LAMP2. Representative images are shown with the individual stainings presented in the inset boxes. The yellow spots in the overlaid images represent lipid segregation that occurred in the lysosomes. Scale bar, 50 *μ*m

In cholesterol efflux fluorometric assays that were performed, the percentage of cholesterol efflux after BBR-SDT was significantly greater than that observed for the control groups (Figure 7a and Supplementary Figure S3a). To further verify the function of autophagy on cholesterol efflux, autophagy-related protein, Atg5, was knocked down with small interfering RNA (siRNA) (Figure 7b). Dilox-LDL, an ox-LDL labeled with a fluorescent probe, was then used to detect aggregates of lipid droplets and efflux. As shown in Figure 7c and Supplementary Figure S3b, ox-LDL content was significantly lower in the BBR-SDT-treated group than in the control groups, and these results suggest that BBR-SDT accelerates the clearance of lipid droplets of foam cells. In contrast, these effects were abolished by NAC and 3MA, implying that ROS and autophagy play important roles in cholesterol efflux.

Roles for ABCA1 and ABCG1 in BBR-SDT-induced cholesterol efflux in foam cells were also examined. The level of ABCA1 post-BBR-SDT was found to increase (Figure 7d and Supplementary Figure S3c), with the greatest increase observed 6 h post-BBR-SDT. Meanwhile, the level of ABCG1 remained stable. Following the knockdown of *Atg5*, no significant effect on the expression of either ABCA1 or ABCG1 was observed in the BBR-SDT groups treated with siRNA or the control groups (Figure 7e).

Lysosomal lipid deposition was observed in control cells, whereas inhibition of autophagy following treatment with 3MA and siRNA targeting *ATG5*, or inhibition of ROS with NAC, both resulted in a profound increase in lysosomal lipid deposition, with larger lysosome marking lysosomal storage disorders. In contrast, induction of autophagy in BBR-SDT- or rapamycin-treated foam cells resulted in a dramatic attenuation of lysosomal lipid deposition, and the presence of smaller lysosomes was consistent with newly reformed functional lysosomes. Taken together, these results indicate that induction of ROS generation and autophagy are pivotal in regulating lipid deposition in macrophages via lysosome/autophagolysosome-mediated degradation.

## Discussion

The results of the present study demonstrate that induction of autophagy and an override of autophagy resistance occurred in BBR-SDT-treated THP-1 macrophages, peritoneal macrophages, and derived foam cells. In addition, key molecules in the PI3K/AKT/mTOR signaling pathway were found to be regulated by BBR-SDT, whereas cholesterol efflux was enhanced following BBR-SDT. These effects were blocked when the generation of ROS and the autophagy process were inhibited. Thus, the induction of lipid release via efficient cholesterol efflux following BBR-SDT indicates that BBR-SDT may represent a promising treatment regimen for atherosclerosis.

A key feature of atherosclerosis is the accumulation of macrophages within lesions. This drives the local inflammatory process and promotes plaque rupture and thrombosis.^45^ Accordingly, targeting of macrophages represents an efficient strategy for the prevention and reversal of atherosclerosis. In the very early stages of atherogenesis, many macrophages and dendritic-like cells have membrane-bound lipid droplets present in their cytoplasm. These lipid-loaded cells are called ‘foam cells', and they form as a result of modified LDL uptake by scavenger receptors.^4^ There are many *in vitro* models of foam cell formation, including treatment of macrophages with Ac-LDL (a modified LDL) or other modified lipoproteins such as ox-LDL.^17^ However, the latter can form *in vivo* as well, and may accumulate under pathological conditions.^17^ In the present study, both macrophages and macrophage-derived foam cells were selected as models for our studies. The latter were generated with the treatment of the macrophages with ox-LDL.^46^ Autophagy resistance was observed 6 h after the addition of ox-LDL, and impaired autophagy efflux led to accumulation of p62. Therefore, this timeframe was considered optimal for inducing autophagy-deficient foam cell formation. Moreover, this result is in agreement with the results of a previous study where reduced autophagy was observed following exposure to ox-LDL.^47^ Vascular smooth muscle cells have also been shown to give rise to a significant number of foam cells.^48^

SDT is an intriguing new approach for cancer therapy, and in recent years, it has also been investigated as a strategy for atherosclerosis treatment.^22,49^ It has been demonstrated that ROS are one of the main mediators or inducers of the biological effects of SDT treatments, whether for cancer or atherosclerosis.^28, 43, 44, 50^ In the present study, ROS generation was detected in both the BBR-SDT-treated macrophages and foam cells by flow cytometry. ROS have been shown to be a stress factor for apoptosis and autophagy.^51, 52, 53, 54^ In most SDT-related studies of atherosclerosis, ROS have played a desirable role in macrophage elimination by inducing apoptosis, and this may be the main cause of stabilized plaques.^22, 49^ BBR-SDT-induced autophagy was also attributed to ROS in the macrophages and derived foam cells used in the present study, while pretreatment with the ROS scavenger, NAC, attenuated these events. These results indicate that BBR-SDT-induced ROS generation may activate autophagy in both macrophages and derived foam cells.

Sonosensitizers derived from natural products have been found to play an important role in SDT as well. Previously, we showed that certain compounds in Chinese herb products exert potent sonodynamic activity, including hypericin, curcumin, and a derived derivative of curcumin.^29, 38, 40^ The use of sonosensitizers has advantages, including use of a lower dose and greater stability to induce a decrease in cell viability. However, the clinical translation of sonosensitizers is currently limited by their lipophilic basis and an absence of their application in the clinic. Berberine, an alkaloid that was originally isolated from *Coptischinensis*, was shown to exhibit sonodynamic activity in both macrophages and derived foam cells in combination with ultrasound irradiation. In the present study, the ability to induce autophagy by BBR-SDT was achieved following an optimization of berberine doses in both macrophages and derived foam cells. Moreover, increased conversion of LC3-I to LC3-II, decreased levels of p62, MDC staining, and electron microscopy data provided further support for the use of BBR-SDT. Interestingly, application of berberine and ultrasound alone under the optimized conditions did not induce autophagy, and these results differ from those previously obtained regarding the capacity for berberine alone to induce autophagy.^55^ The application of varying doses of berberine and the use of different solvents may account for this difference in results. In the present study, the conversion of LC3-I to LC3-II increased in a time-dependent manner following BBR-SDT in both the macrophages and foam cells, although the peak time differed between the two models. In a study by Su *et al.*,^32^ 0.5 h was the most obvious time point for the induction of autophagy following protoporphyrin IX-SDT, and this time point differs markedly from our results. On the basis of these data, it is hypothesized that the use of different types of sonosensitizers and cell lines can lead to differences in the peak time observed for the induction of autophagy.

Understanding the hierarchy between regulation of ROS and apoptosis or autophagy is essential for clarifying the molecular mechanisms involved in SDT. It is known that the PI3K/AKT/mTOR signaling pathway plays an important role in cell survival, proliferation, and metabolism; and suppression of this signaling pathway can lead to cell survival or cell death via autophagy or apoptosis, respectively.^56, 57^ In the present study, BBR-SDT reduced the activation of AKT (phospho-Akt^Ser473^), as well as mTOR (phospho-mTOR^Ser2448^), in the macrophages examined. Furthermore, autophagy that was enhanced by BBR-SDT was abolished in the presence of the PI3K inhibitor, LY294002. Meanwhile, BBR-SDT showed effects similar to that of the mTOR-specific inhibitor, rapamycin, in mediating an induction of autophagy in macrophages. Consistent with these observations, BBR-SDT was also found to lower the levels of phosphorylated Akt^Ser473^ and mTOR^Ser2448^ in ox-LDL-induced foam cells. Overall, these observations indicate that suppression of the PI3K/AKT/mTOR signaling pathway is a characteristic of BBR-SDT-induced autophagy.

Autophagy is well known for its cytoprotective and cytotoxic effects. In addition, autophagy appears to enhance the hydrolysis of stored cholesterol droplets in macrophages, thus facilitating cholesterol efflux.^58^ There are several lines of evidence in the present study that indicate that BBR-SDT-induced autophagy controls autophagy-dependent cholesterol efflux. First, Dilox-LDL stainings demonstrated that 6 h post-BBR-SDT, the levels of ox-LDL had decreased compared with the control groups. Moreover, these effects were blocked by 3MA. These results indicate the positive effect of autophagy on Dilox-LDL clearance. Second, increased levels of ABCA1, a protein that has been closely linked to cholesterol efflux, were observed 6 h post-BBR-SDT, and this increase was reversed when levels of ATG5 were reduced. Both ABCA1 and ABCG1 have been found to promote cholesterol efflux via their interactions with high-density lipoproteins (HDLs), although a significant change in ABCA1 levels has been observed while the levels of ABCG1 remained unchanged.^61, 62^ Our results are consistent with the latter. Third, in co-localization analyses of Bodipy and LAMP2 6 h post-BBR-SDT, fewer lipid droplets were observed while the number of lysosomes increased. These results were reversed in the presence of 3MA and siRNA targeting *Atg5*,thereby indicating that autophagy resistance can interfere with the effects of cholesterol efflux via BBR-SDT. Moreover, an increase in autophagy in response to BBR-SDT may represent a defense mechanism by which cholesterol efflux is enhanced in foam cells.

**Figure 8:**
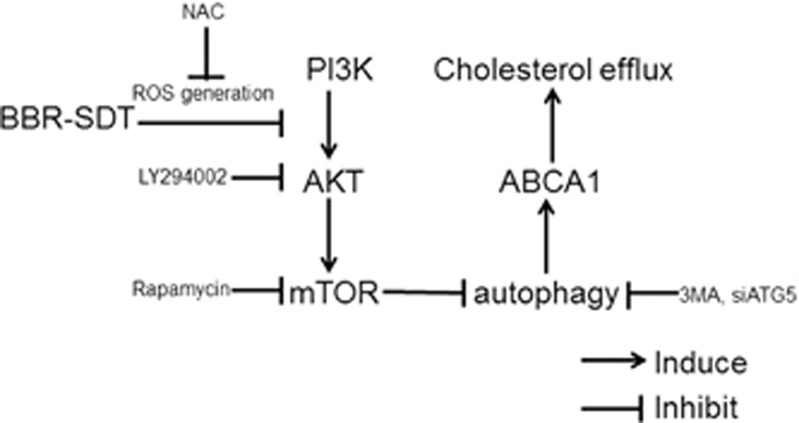
Schematic diagram showing the proposed mechanism underlying BBR-SDT-induced autophagy and cholesterol efflux in THP-1 macrophages and derived foam cells

As illustrated schematically in Figure 8, the findings from the present study demonstrate that the application of BBR-SDT to both “normal” macrophages and lipid-loaded macrophages (e.g., foam cells) represents an effective means of enhancing ROS generation which subsequently induces autophagy via suppression of the PI3K/AKT/mTOR pathway and promotes cholesterol efflux. It is anticipated that further investigation of these findings in pre-clinical and clinical settings could lead to the development of SDT as an effective therapy for atherosclerosis.

## Materials and Methods

### Cell culture

Human THP-1 monocytes (American Type Culture Collection, Manassas, VA, USA) were cultured in RPMI 1640 medium (HyClone, Logan, UT, USA) supplemented with 10% fetal bovine serum (FBS;HyClone), 20 *μ*g/ml penicillin, and 20 *μ*g/ml streptomycin (Sigma-Aldrich, St. Louis, MO, USA). The cells were maintained at 37 °C, 5% CO_2_and the medium was replaced every 2–3 days. To induce differentiation of the macrophages, the THP-1 monocytes were incubated with 100 ng/ml phorbol-12-myristate-13-acetate (PMA, EMD Biosciences, La Jolla, CA, USA) in RPMI 1640 medium containing 10% FBS for 72 h, at a density of 1.0 × 10^5^ cells/ml. The THP-1 macrophages were subsequently transformed into THP-1 macrophage-derived foam cells by adding 50 *μ*g/ml oxidized-low-density lipoprotein (ox-LDL, Yiyuan Biotechnologies, Guangzhou, China) in serum-free RPMI 1640 medium containing 0.3% bovine serum albumin (BSA) for 12 h.

### Ultrasound system

The ultrasound system employed was provided by the Condensed Matter Science and Technology Institute of the Harbin Institute of Technology (Harbin, China), and has been described previously.^29, 38^

### SDT protocol

BBR was obtained from Chengdu Must Bio-Technology Co. (Chengdu, China) and was stored in ddH_2_O as a 1 mg/ml stock solution at 4 °C in the dark. When THP-1 macrophages reached an exponential phase, they were collected, differentiated, and randomly divided into four groups: (1) control, (2) ultrasound alone, (3) berberine alone, and (4) BBR-SDT. For the berberine and BBR-SDT groups, the cells were incubated with the indicated doses of BBR for 4 h in RPMI 1640 medium containing FBS. The control and ultrasound alone groups received an equivalent volume of medium instead of BBR. The cells in the ultrasound and BBR-SDT groups were exposed to ultrasound at a frequency of 1.0 MHz for the indicated intensities. After the treatments, the cells were carefully washed once in phosphate-buffered saline (PBS), were cultured in fresh medium for a few hours, and then were subjected to different analyses.

Depending on the experiments performed, 3MA (Sigma-Aldrich), LY294002 (Selleck Chemicals, Houston, TX, USA), rapamycin (Rapa; Selleck Chemicals), or hydroxychloroquine (Selleck Chemicals) were added to the culture medium in combination with BBR for 4 h. When appropriate, NAC was added to the culture medium 30 min prior to the addition of BBR-SDT.

### Cell viability assay

CCK-8 assays were performed as previously described^29, 38^ Briefly, THP-1 macrophages and foam cells were seeded in 96-well cell culture plates and treated as indicated. The medium in each well was then replaced with fresh medium (without FBS) containing CCK-8 reagent (Beyotime, Beijing, China) in a 9:1 ratio. After 2 h, absorption values at 450 nm were measured for each well with a microplate reader (Varian Australia Pty Ltd., Australia). Each sample was assayed in triplicate.

### Detection of intracellular ROS

ROS were measured by using DCFH-DA, a reagent which is de-esterified intracellularly and undergoes oxidation to form a highly fluorescent molecule, 2',7'-dichlorofluorescein.^63^ Briefly, treated cells were harvested, washed with PBS, and stained with 20 *μ*M DCFH-DA (Applygen Technologies, Beijing, China) for 20 min at 37 °C in the dark. Fluorescent signals were detected with a FACS Verse flow cytometer (BD, Germany).

### TEM

Cells were processed as previously described^29^ and digital images were obtained with a transmission electron microscope (JEM-1220, Japan).

### Western blotting assay

Western blotting was performed as previously described.^29, 38, 64^ Briefly, denatured protein samples were separated by 10%, 12.5%, or 15% sodium dodecyl sulfate-polyacrylamide gel electrophoresis (SDS-PAGE) and were transferred to 0.45-*μ*m PVDF membranes at 300 mA for 90 min up to 150 min. After blocking the membranes at room temperature for 1 h in 5% low-fat milk powder in Tris-buffered saline-Tween 20, the membranes were probed with primary antibodies and incubated at 4 °C overnight. The primary antibodies used were raised against: LC3B (Cell Signaling Technology, Beverly, MA, USA and Sigma-Aldrich), p62 (Cell Signaling Technology and Abcam, Burlingame, CA, USA), mTOR, p-mTOR, AKT, p-AKT, Atg5 (Cell Signaling Technology), ABCA1 (Abcam), ABCG1 (Santa Cruz Biotechnology, Dallas, TX, USA), and *β*-actin (ZSGB-BIO, Inc., Beijing, China). All of the primary antibodies were diluted 1:1000. After washing, the membranes were incubated with the appropriate horseradish peroxidase-labeled secondary antibodies (diluted 1:1000) for 1 h at room temperature. Bound antibodies were visualized with enhanced chemiluminescence reagents. After a final wash with Tris-buffered saline-Tween 20, multiple images were obtained and the protein bands were quantified by using a Bio-Rad Chemi EQ densitometer and Bio-Rad Quantity One software (BioRad Laboratories, Hercules, CA, USA).

### MDC staining

Autophagic vacuoles were detected by MDC, a phospholipid-specific marker that selectively accumulates in autophagosomes.^42^ Briefly, cells were incubated with MDC (50 *μ*M, Cayman Chemical Co., Ann Arbor, MI, USA) in fresh medium (without FBS) for 30 min in the dark. After washing the samples with PBS, fluorescence microscopy (excitation wavelength (Ex), 372 nm; emission wavelength (Em), 456 nm) was used to detect distinct dot-like structures that represent autophagosomes.^42^

### Oil red O staining

Following BBR-SDT, macrophage-derived foam cells were fixed with 10% formalin for 1 h, were rinsed with 60% isopropanol, and were incubated with freshly filtered 0.5% Oil red O solution for 10 min at 37 °C. The cells were then washed in isopropanol for 10 min, rinsed in distilled water, and hematoxylin was added to label cell nuclei. Images were obtained with a fluorescence microscope and lipid accumulation was examined.

### Localization of Dilox-LDL to nuclei in THP-1 macrophages

THP-1 macrophages were incubated with 10 *μ*g/ml Dilox-LDL (Yiyuan Biotechnologies) at 37 °C for 16 h. The medium was then removed, the cells were washed, and BBR-SDT treatment was administered. After the cells were stained with Hoechst 33258 (Sigma-Aldrich) for 5 min at 37 °C in the dark, images were obtained with a fluorescence microscope.

### Immunofluorescence

Cells were cultured on glass coverslips, were treated as indicated, and were fixed with 4% paraformaldehyde for 30 min. After the coverslips were blocked with 10% normal goat serum for 1 h at room temperature, the cells were incubated with an anti-LC3 antibody or an anti-LAMP2 antibody (1:100, Santa Cruz Biotechnology) overnight at 4 °C. After an incubation with the corresponding secondary antibodies for 1 h at 37 °C, the cells were stained with BODIPY (FL C5-ceramide D-3521, Life Technologies, Invitrogen, Grand Island, NY, USA; stock solution: 1 mg/ml in ethanol, working solution: 10 *μ*g/ml diluted in PBS) in the dark at room temperature for 20 min. A subsequent staining was performed with 1.0 ml of 0.5 *μ*g/ml Hoechst 33258 for 5 min at 37 °C in the dark and the cells were examined with laser scanning confocal microscopy.

### Cholesterol efflux fluorometric assay

The effect of BBR-SDT on cholesterol efflux was measured by using a Cholesterol Efflux Fluorometric assay kit (BioVision, CA, USA) according to the manufacturer's protocol.^65^ Briefly, 50 *μ*l of labeling reagent and 50 *μ*l of equilibration buffer containing reagent A and B were mixed and added to each well (100 *μ*l of mix/well). THP-1 macrophages were then added to each well and were incubated overnight. After 16 h, the cells were washed and the indicated doses of berberine were added. After 4 h, the cells were washed and incubated with HDL (50 *μ*g/well, Yiyuan Biotechnologies) as cholesterol acceptors, and then were exposed to ultrasound. Six hours later, the supernatants were transferred to a white 96-well plate, the cells were solubilized with cell lysis buffer (100 *μ*l), shaken for 30 min, and fluorescence was measured (Ex/Em=482/515 nm). Cholesterol efflux was calculated by dividing the fluorescence intensity of the media by the total fluorescence intensity of the cell lysate of the same treatment + media.

### Transfection of a siRNA targeting *Atg5*

Cells were grown in 35 mm cell culture plates and transfected with siRNA using Opti-MEM media (Invitrogen Life Technologies, Carlsbad, CA, USA) according to the manufacturer's protocol. Briefly, 5 *μ*l of Atg5 siRNA (20 *μ*M) was mixed with Opti-MEM media. Separately, X-tremeGene Transfection Reagent (Roche, Basel, Switzerland) was mixed with Opti-MEM media. The mixtures were combined for 20 min at room temperature before being added to each well. After 6 h, the medium in each well was replaced with antibiotic-free 1640 medium supplemented with 10% FBS for 48 h.

Atg5 siRNA duplexes:

S1 (sense: GCUUCGAGAUGUGUGGUUUTT; antisense: AAACCACACAUCUCGAAGCTT)

S2 (sense: CCAUCAAUCGGAAACUCAUTT; antisense: AUGAGUUUCCGAUUGAUGGTT)

S3 (sense: GCAGUGGCUGAGUGAACAUTT; antisense: AUGUUCACUCAGCCACUGCTT).

### Statistical analysis

All of the experiments described were performed independently at least three times. Data were analyzed using one-way analysis of variance (ANOVA) and are presented as the mean±S.D. A *P*-value less than 0.05 was considered statistically significant.
