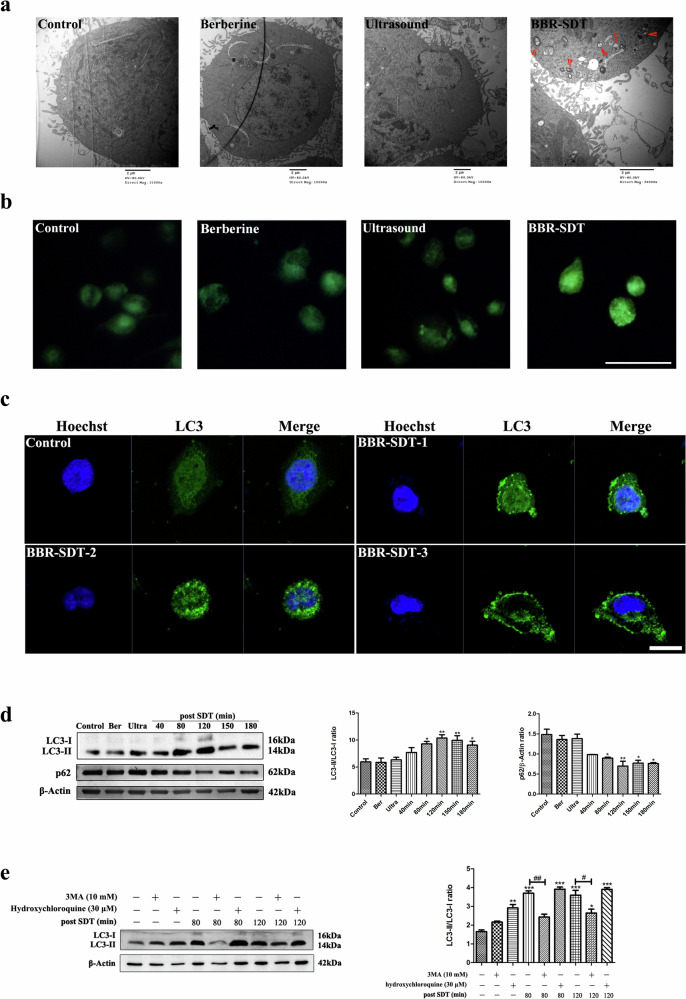# Correction: Berberine-sonodynamic therapy induces autophagy and lipid unloading in macrophage

**DOI:** 10.1038/s41419-026-08693-2

**Published:** 2026-04-02

**Authors:** Jiayuan Y. Kou, Ying Li, Zhaoyu Y. Zhong, Yueqing Q. Jiang, Xuesong S. Li, Xiaobo B. Han, Zhongni N. Liu, Ye Tian, Liming M. Yang

**Affiliations:** 1 Department of Pathophysiology, Key Laboratory of Cardiovascular Pathophysiology, Harbin Medical University, Harbin, PR China; 2 Department of Oncology, The First Affiliated Clinic College of Harbin Medical University, Harbin, PR China; 3 Division of Cardiology, The First Affiliated Hospital, Harbin Medical University, Harbin, PR China

Correction to: *Cell Death & Disease* 10.1038/cddis.2016.354, published online 19 January 2017

In the original version of this article, Figure 2b contained an error. Due to a typesetting oversight during figure assembly, the representative images for the Control and Berberine groups were inadvertently duplicated. The corrected Figure 2b, which provides the accurate representative images, is shown below. This correction does not affect the conclusions of the paper. The authors apologize for this error.

**Figure 2 Original** 
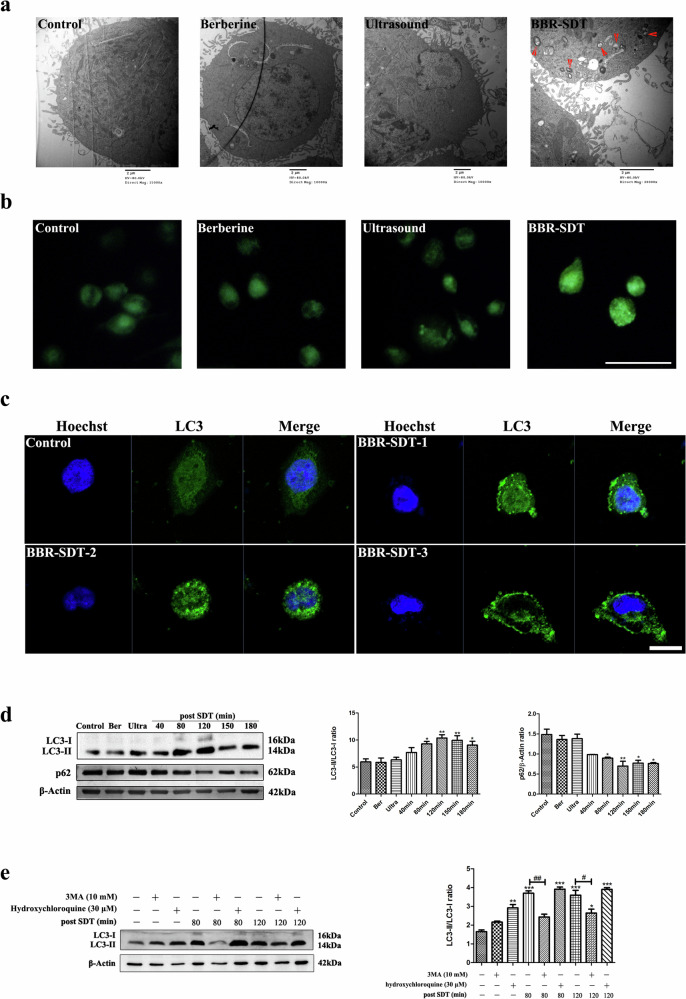


**Figure 2 Amended**