# Supplementary material for: Berberine-sonodynamic therapy induces autophagy and lipid unloading in macrophage
Source: Cell Death Dis. 2017 Jan 19;8(1):e2558–. doi: 10.1038/cddis.2016.354 (PMC5386349; doi:10.1038/cddis.2016.354)
Supplement: Supplementary Information [file cddis2016354x1.docx]

**Berberine-sonodynamic therapy induces autophagy and lipid unloading in macrophage**

Running title: Sonodynamic therapy induces autophagy and lipid unloading

Jiayuan Kou^1,4^, Ying Li^2,4^, Zhaoyu Zhong^1^, Yueqing Jiang^1^, Xuesong Li^1^, Xiaobo Han^1^, Zhongni Liu^1^, Ye Tian*^1,3^, Liming Yang*^1^

**Supplementary Methods**

**Cell culture.** Peritoneal macrophages were recruited from C57BL/6 (B6) mice by injecting 2.5 ml 10% thioglycollic acid broth 3 days before collecting the cells with icecold phosphate-buffered saline (PBS). Then, cells were rested on 35mm dishes in RPMI with 10 % fetal bovine serum (FBS) for 24 hrs before experiments. Peritoneal macrophages were transformed into peritoneal macrophage-derived foam cells by adding 50 μg/mL ox-LDL (oxidized-low density lipoprotein, Yiyuan Biotechnologies, Guangzhou, China) for 12 h in serum-free RPMI 1640 medium containing 0.3% BSA.

**Mice.** C57BL/6 (B6) mice were purchased from HFK Bioscience Co., Ltd. (Beijing, China) and housed in the animal facilities of the Harbin Medical University. According to protocols approved by the Institutional Animal Care and use Committee at the Institute of Genetics and Developmental Biology**,** all experimental procedures involved were performed.

**Ultrasound exposure system.** The ultrasound exposure system was provided by Condensed Matter Science and Technology Institute of the Harbin Institute of Technology (Harbin, China), as previously described.^29, 38^

**SDT protocol.** BBR was obtained from Chengdu Must Bio-Technology Co., Ltd and was stored in ddH_2_O as 1 mg/mL stock solution at 4 °C in the dark.
Peritoneal macrophages were collected and randomly divided into four groups: 1) Control, 2) Ultrasound alone, 3) Berberine alone, and 4) BBR-SDT (means berberine under ultrasound irradiation). For the berberine and BBR-SDT groups, the cells were incubated with indicated doses of BBR for a drug loading time of 4 hours in FBS-loaded RPMI 1640 medium. Instead of BBR, an equivalent volume of medium was used in the control and ultrasound alone groups. The cells in the ultrasound and BBR-SDT groups were exposed to ultrasound at a frequency of 1.0 MHz and of the indicated intensity. After the treatments, the cells were carefully washed once in PBS, cultured in fresh medium for a few hours and then subjected to different analyses.

Depending on the experiments performed, 3-methyladenine (3MA, Sigma-Aldrich Co., St Louis, MO, USA) was added to the culture medium together with BBR loading for 4 hours. N-acetyl-cysteine (NAC) was added to the culture medium half an hour before BBR-SDT.

**Cell viability assay.** CCK-8 assay was performed as previously described.^29, 38^ In briefly, peritoneal macrophages and foam cells were seeded in 96-well cell culture plate, after treatment, the medium within cell culture plate was placed by fresh medium (without FBS) 100 µL per well containing CCK-8 (Beyotime, Beijing, China) (the ratio of medium and CCK-8 volume was 9:1). After incubation for 2 h, the absorption at 450 nm of each well was measured using a microplate reader (Varian Australia Pty Ltd., Australia). Each assessment was performed in triplicate.

**Detection of intracellular ROS.** ROS measurement was performed by using DCFH-DA, which was de-esterified intracellularly and became highly fluorescent 2’,7’ -dichlorofluorescein upon oxidation.^63^ In briefly, the treated cells were harvested, washed with PBS, and then stained with 20 *µ*M DCFH-DA (Applygen Technologies Co. Ltd., Beijing, China) for 20 min at 37 °C in the dark post BBR-SDT. The fluorescent signal produced was analyzed by using flow cytometer FACS Verse (BD, Germany).

**Transmission electron microscopy examination.** The treated cells were processed as previously described.^29^ Digital images were obtained with a transmission electron microscope (JEM-1220, Japan).

**Western blotting assay.** Western blotting was performed as previously described.^29, 38, 64^ Briefly, denatured proteins samples of different molecular weight were separated in 10%, 12.5% or 15% sodium dodecyl sulfate-polyacrylamide gel electrophoresis (SDS-PAGE) and transferred into 0.45-*μ*m PVDF membrane at 300 mA for 90 min and 150 min. After incubating at room temperature for 1h in 5% low-fat milk powder in tris-buffered saline-Tween 20 (TBST) for blocking, the membranes were probed with primary antibodies against LC3B (Cell Signaling Technology, Inc., USA and Sigma-Aldrich Co., St Louis, MO, USA), p62 (Cell Signaling Technology and Abcam, Burlingame, CA, USA), ABCA1 (Abcam), ABCG1(Santa Cruz Biotechnology, Santa Cruz, CA, USA), and β-Actin (ZSGB-BIO, Inc., Beijing, China, all primary antibodies above were diluted with 1:1000) at 4 °C overnight. After washing, the membranes were incubated with HRP-labeled secondary antibodies (all secondary antibodies above were diluted with 1:1000) for 1h at room temperature. The immune complexes were detected with enhanced chemiluminescence reagents following washing with TBST. Multiple images were then taken, and the volumes of protein bands were quantified by Bio-Rad Chemi EQ densitometer and Bio-Rad Quantity One software (BioRad Laboratories, Hercules, USA).

**Oil red O staining.** After BBR-SDT, cells were fixed with 10% formalin for 1h, followed by rinse with 60% isopropanol and incubation with fresh-filtered 0.5% Oil red O solution for 10 min at 37 °C. For analysis, the cells were washed in isopropanol for 10 min, rinsed in distilled water, and hematoxylin was introduced to label the cell nuclei. Images of cells were captured using a fluorescence microscope to evaluate the characteristic lipid accumulation in macrophage-derived foam cells.

**Co-localization of Dil ox-LDL with nuclei in peritoneal macrophages.** Peritoneal macrophages were incubated with 10 mg/mL Dil ox-LDL (Yiyuan Biotechnologies, Guangzhou, China) at 37 °C for 16 h. After the medium was removed, cells were washed and under BBR-SDT treatment. After that, the cells were stained with Hochest 33258 (Sigma-Aldrich Co., St Louis, MO, USA) for 5 minutes at 37 °C in the dark. Images of cells were captured using a fluorescence microscope.

**Cholesterol efflux fluorometric assay.** Effect of BBR-SDT on cholesterol efflux was measured by using Cholesterol efflux fluorometric assay kit (BioVision, Inc., CA, USA) according to manufacturer’s protocol.^65^ In briefly, Premix 50 μL of Labeling Reagent and 50 μL of Equilibration Buffer containing Reagent A and B/well just before using. Peritoneal macrophages were added with 100 μL of mix/well and incubated overnight. After 16 h, cells were washed and added indicated doses of berberine for 4 h. After washing, cells were incubated with HDL (50 μg/well, high density lipoprotein, Yiyuan Biotechnologies, Guangzhou, China) as cholesterol acceptors, and then exposed to ultrasound. 6h later, transfer supernatant to a 96-well plate (white plate) and measure the fluorescence (Ex/Em=482/515 nm). Solubilize the cell monolayer by adding 100 μL of Cell Lysis Buffer. After shaking for 30 min, measure the fluorescence (Ex/Em=482/515 nm). Cholesterol efflux of the treatments is calculated by dividing the fluorescence intensity of the media by total fluorescence intensity of the cell lysate of the same treatment & media.

**Statistical analysis.** All experiments were performed independently at least three times. The data were analyzed using one-way ANOVA and are presented as means ± standard deviation (SD). A P-value less than 0.05 was considered statistically significant.

**Figure Legends**

**Supplementary Figure 1 Berberine induced autophagy of peritoneal macrophages upon ultrasound exposure.** (a) The effects of berberine with or without ultrasound irradiation on the viability of peritoneal macrophages with the application of: (1) different concentrations of berberine, and (2) different concentrations of BBR-SDT (0.4 W/cm^2^ ultrasound irradiation) as indicated. Cell viability was analyzed in CCK-8 assays and the data are presented as the mean ± SD. (^*^*P* < 0.05, ^***^*P* < 0.001 vs. Control). (b) Levels of LC3-I, LC3-II, and p62 in peritoneal cells post-BBR-SDT were analyzed by Western blotting. Quantitation of the LC3-II/LC3-I ratios and p62 levels are presented to the right as the mean ± SD (n = 3; ^*^*P* < 0.05 and ^**^*P* < 0.01 vs. Control). (c) Ultrastructural changes in untreated (control), and BBR-SDT-treated peritoneal macrophages were observed by TEM at 120 min post-BBR-SDT. Red arrows indicate autophagosomes. Scale bar=2 μm. (d) Levels of LC3-I, LC3-II, and p62 in peritoneal cells post-BBR-SDT were analyzed by Western blotting. Quantitation of the LC3-II/LC3-I ratios and p62 levels are presented to the right as the mean ± SD (n = 3; ^*^*P* < 0.05 vs. Control). (e) Western blot analysis and quantification of fold-change in LC3-II/LC3-I ratios and p62 levels in peritoneal cells with and without pretreatment with NAC or 3MA and post-BBR-SDT (^*^*P* < 0.05 vs. Control; ^#^*P* < 0.05 vs. BBR-SDT groups). (f) The relative fluorescence intensity for ROS generation detected in peritoneal macrophages with or without the ROS scavenger, NAC (1 mM), as determined by flow cytometry following DCFH-DA staining (n = 3; berberine autofluorescence).

**Supplementary Figure 2 BBR-SDT similarly induced autophagy in peritoneal macrophage-derived foam cells induced by ox-LDL.** (a) Representative images of peritoneal macrophage morphology following incubation with ox-LDL for 12 h are shown. Oil Red O staining was performed to examine foam cell formation. Scale bar, 100 μm. (b) The effects of berberine with or without ultrasound irradiation on the viability of peritoneal macrophage-derived foam cells with the application of: (1) different concentrations of berberine, and (2) different concentrations of BBR-SDT (0.4 W/cm^2^ ultrasound irradiation) as indicated. Cell viability was analyzed in CCK-8 assays and the data are presented as the mean ± SD. (^***^*P* < 0.001 vs. Control). (c and d) Levels of LC3-I, LC3-II, and p62 in foam cells post-BBR-SDT were analyzed by Western blotting. Quantitation of the LC3-II/LC3-I ratios and p62 levels are presented to the right as the mean ± SD (n = 3; ^*^*P* < 0.05, ^**^*P* < 0.01 and ^***^*P* < 0.001 vs. Control). (e) Ultrastructural changes in foam cells treated with ox-LDL were observed by TEM post-BBR-SDT. Black arrows indicate lipid droplets, red arrows indicate autophagosomes. Scale bar, 2 μm. (f) The relative fluorescence intensity for ROS generation detected in foam cells with or without the ROS scavenger, NAC (1 mM), as determined by flow cytometry following DCFH-DA staining (n = 3; berberine autofluorescence). (g) Western blot analysis and quantification of fold-change in LC3-II/LC3-I ratios and p62 levels in foam cells with and without pretreatment with NAC or 3MA and post-BBR-SDT (^*^*P* < 0.05 vs. Control; ^#^*P* < 0.05 vs. BBR-SDT groups).

**Supplementary Figure 3 BBR-SDT induced cholesterol efflux in peritoneal macrophage-derived foam cells.** (a) Peritoneal macrophages were incubated with Labeling Media and then were treated with various HDL (50 μg) as cholesterol acceptors to induce cholesterol efflux post BBR-SDT. The data are expressed as % efflux detected 6 h post-BBR-SDT (n=3; ^***^*P* < 0.001 vs. Control). (b) [Fluorescence microscopy](http://www.baidu.com/link?url=0YFOgBOudN8djESxmyjkb_6JJTjmxK5R-29bfeRa2mJjIvXOpWBfgVFgERssL8uknohIlDic4V5MWSsNiP82j4G9oKYcg-Hx-vzDLRdAjI9c1WM-4i1D3bQPOlysLosa) images of peritoneal macrophages that were pretreated with 3MA or NAC, were subjected to BBR-SDT, and then were incubated with Dil ox-LDL for 6 h. Red: Dil ox-LDL derivatives, blue: Hoechst-stained nuclei. Scale bar, 200 μm. (c) Extracts of foam cells were subjected to Western blotting to analyze levels of ABCA1, ABCG1, and β-actin post-BBR-SDT. Quantification of these levels are shown to the right as mean ± SD values (n = 3; **P* < 0.05 vs. Control).
