# Supplementary figures and images for: Berberine-sonodynamic therapy induces autophagy and lipid unloading in macrophage
Source: Cell Death Dis. 2017 Jan 19;8(1):e2558–. doi: 10.1038/cddis.2016.354 (PMC5386349; doi:10.1038/cddis.2016.354)

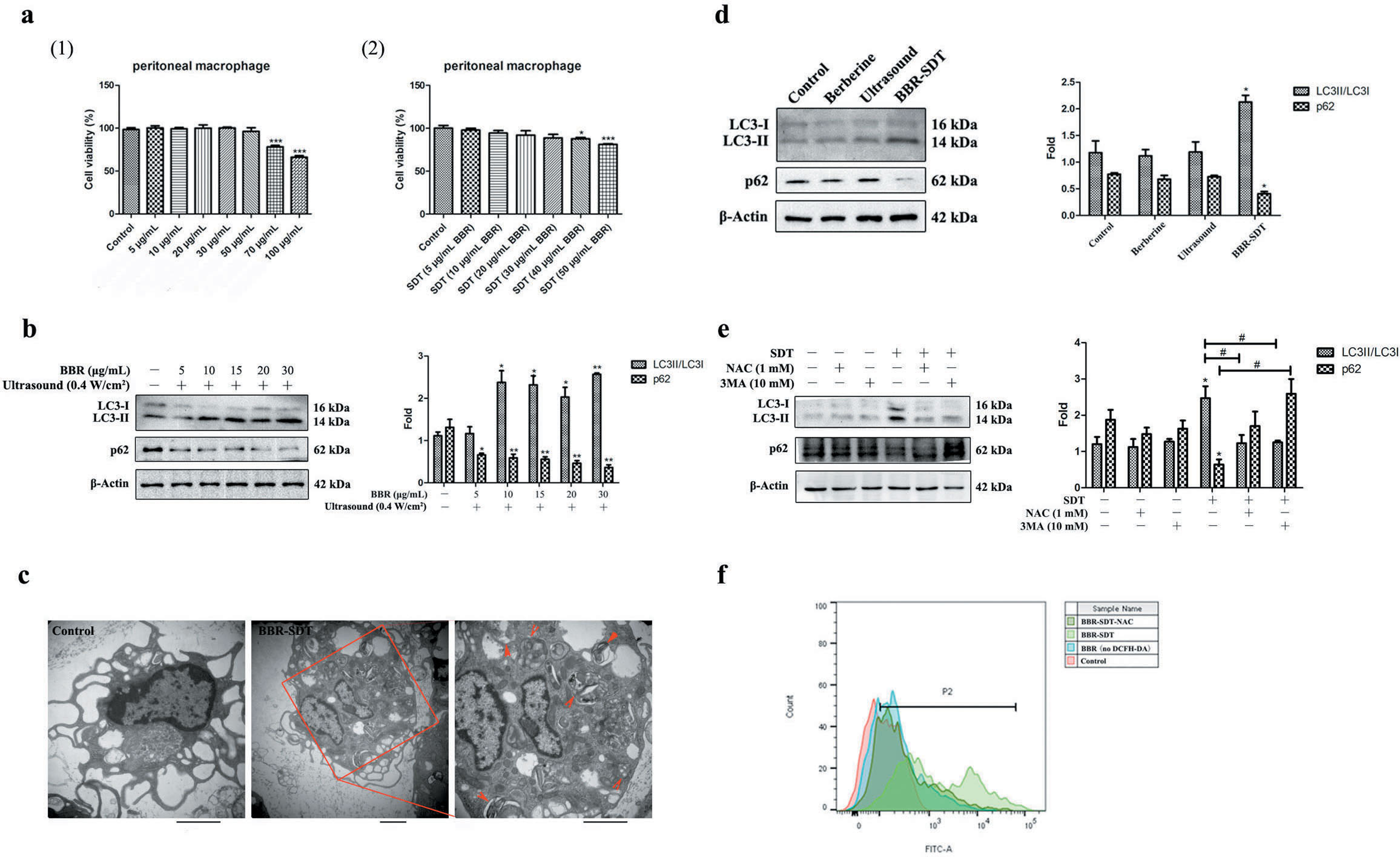

Supplement: Supplementary Figure 1 [file cddis2016354x2.tif]

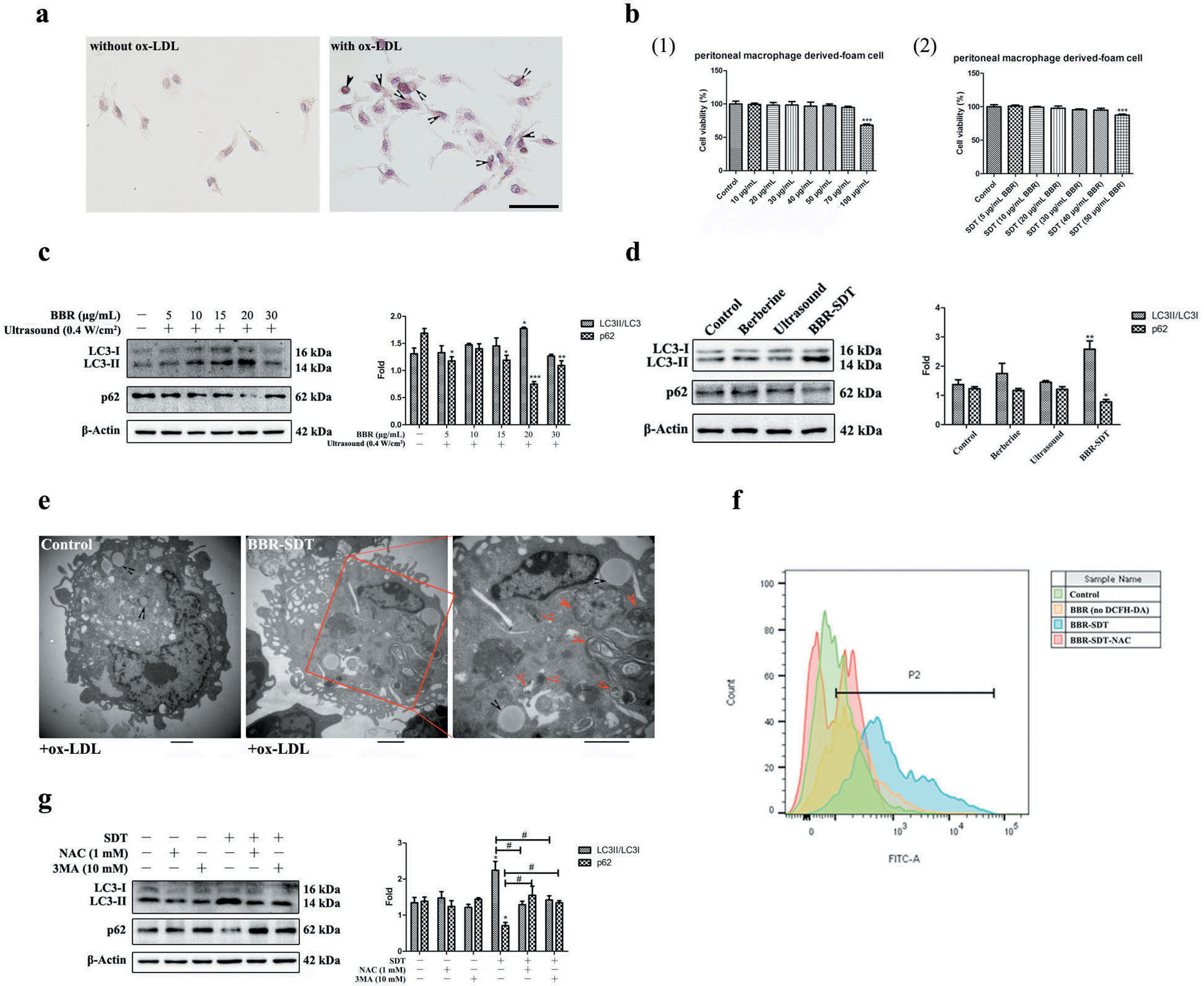

Supplement: Supplementary Figure 2 [file cddis2016354x3.tif]

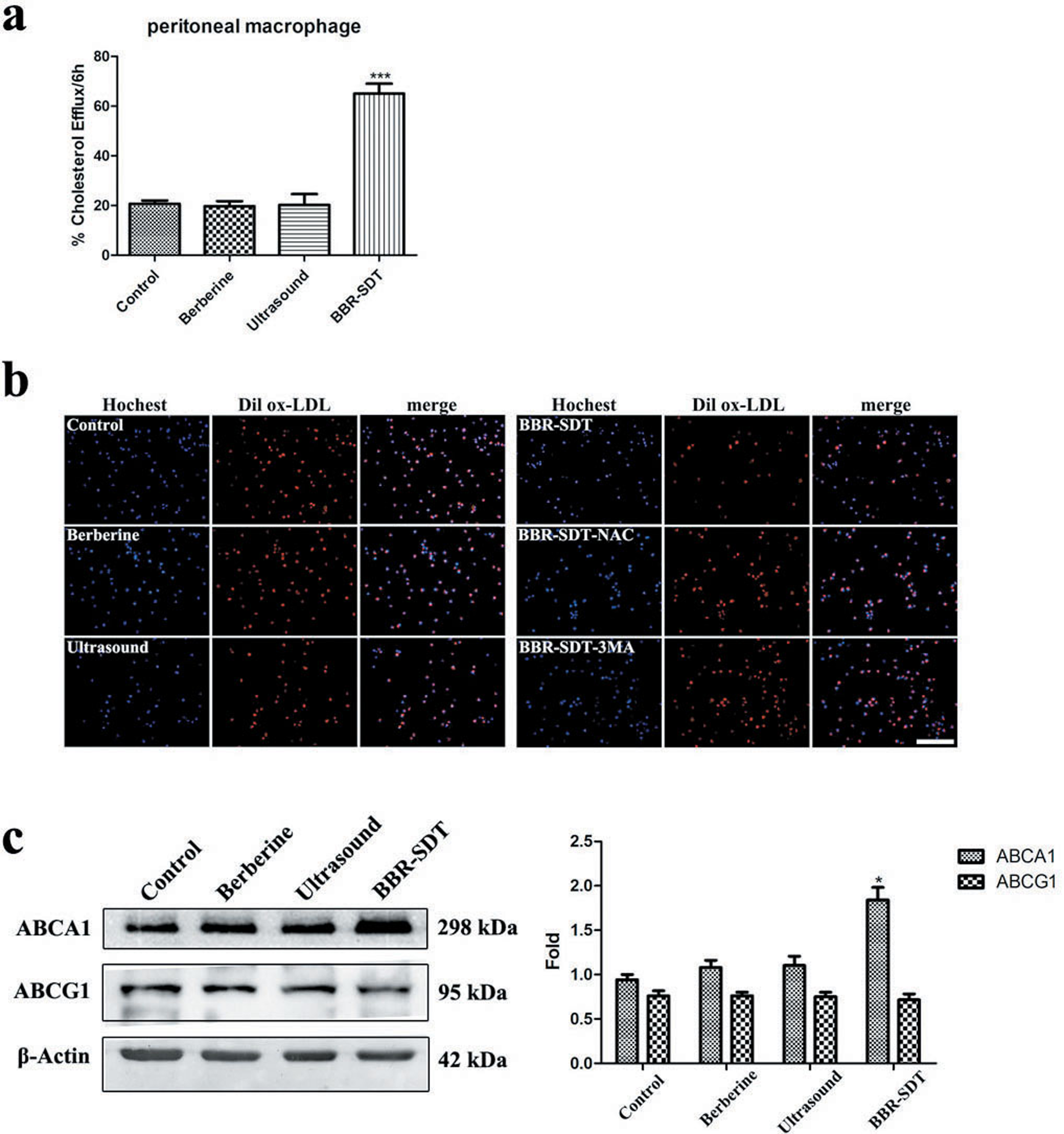

Supplement: Supplementary Figure 3 [file cddis2016354x4.tif]
